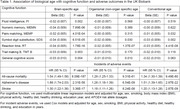# Plasma‐based Brain Age as a Biomarker for Cognitive Health and Risk of Brain‐Related Diseases

**DOI:** 10.1002/alz70856_103849

**Published:** 2025-12-24

**Authors:** Biqi Wang, Huitong Ding, Derek Qi, Mayra S. Tisminetzky, Joanne M Murabito, Honghuang Lin

**Affiliations:** ^1^ University of Massachusetts Medical School, Worcester, MA, USA; ^2^ Boston University Chobanian & Avedisian School of Medicine, Boston, MA, USA; ^3^ Framingham Heart Study, Framingham, MA, USA; ^4^ University of Massachusetts Chan Medical School, Worcester, MA, USA; ^5^ Boston University School of Medicine, Boston, MA, USA

## Abstract

**Background:**

The study of brain aging is crucial for understanding the processes that drive cognitive decline and the development of neurodegenerative diseases. It helps identify early markers of brain health, enabling timely interventions to slow or prevent age‐related neurological conditions.

**Method:**

We included participants from the UK Biobank who had Olink proteomics data (2006‐2010). Brain‐specific proteins were defined as those with expression levels at least four times higher in the brain than in other organs. Proteins were used to calculate brain age, non‐organ‐specific age, and conventional proteomic age by LASSO regression models. The association of these biological ages with cognitive function was tested using multivariable adjusted linear regression models. We further examined their association with incidental diseases (e.g., Alzheimer's disease (AD)) by Cox proportional hazards models. We replicated our findings in the Framingham Heart Study (FHS), which utilized the SOMAscan proteomics platform.

**Result:**

This study included 53,005 participants from the UK Biobank (mean age 57±8 years old, 54% women). The brain age was highly correlated with chronological age (*r* = 0.72). As shown in the Table, accelerated brain aging was associated with lower cognitive scores. Our findings suggest that accelerated brain aging was associated with an increased risk of AD (HR, 95% CI: 1.88 (1.74‐2.03)) and stroke (HR, 95% CI: 1.30 (1.22‐1.38)). It is important to highlight that their effect sizes were larger than those of organismal aging and conventional aging. In the replication cohort of the FHS (*n* =  1,913, mean age 55±10 years old, 54% women), we found that accelerated brain aging was associated with lower attention and concentration performance (effect size = ‐0.05, P value = 0.03) as well as an increased risk of stroke (HR, 95% CI: 1.19 (1.03‐1.36)).

**Conclusion:**

Our findings suggest that brain aging is associated with cognitive performance and increased risk of brain‐related disease in two longitudinal cohorts utilizing different proteomics platforms. Our findings highlight the potential of identifying novel plasma‐based biomarkers for monitoring cognitive health and identifying individuals at risk of developing brain‐related diseases.